# A Canonical Correlation Analysis of AIDS Restriction Genes and Metabolic Pathways Identifies Purine Metabolism as a Key Cooperator

**DOI:** 10.1155/2016/2460184

**Published:** 2016-07-04

**Authors:** Hanhui Ye, Jinjin Yuan, Zhengwu Wang, Aiqiong Huang, Xiaolong Liu, Xiao Han, Yahong Chen

**Affiliations:** ^1^The United Innovation of Mengchao Hepatobiliary Technology Key Laboratory of Fujian Province, Mengchao Hepatobiliary Hospital of Fujian Medical University, Fuzhou 350025, China; ^2^Infectious Diseases Hospital of Fuzhou, Fuzhou 350025, China; ^3^Biotechnology Research Institute, Chinese Academy of Agricultural Sciences, Beijing 100081, China

## Abstract

Human immunodeficiency virus causes a severe disease in humans, referred to as immune deficiency syndrome. Studies on the interaction between host genetic factors and the virus have revealed dozens of genes that impact diverse processes in the AIDS disease. To resolve more genetic factors related to AIDS, a canonical correlation analysis was used to determine the correlation between AIDS restriction and metabolic pathway gene expression. The results show that HIV-1 postentry cellular viral cofactors from AIDS restriction genes are coexpressed in human transcriptome microarray datasets. Further, the purine metabolism pathway comprises novel host factors that are coexpressed with AIDS restriction genes. Using a canonical correlation analysis for expression is a reliable approach to exploring the mechanism underlying AIDS.

## 1. Introduction

Human immunodeficiency virus (HIV) is the basis for acquired immune deficiency syndrome (AIDS) pathogenesis and destroys the lymphoid system with prodigious replicates, which reduces a patient's ability to survive. Since HIV was identified in the 1980s, this pathogen has taken more than 10 million people's lives throughout the world. Researchers have developed considerable information on HIV involving immunology, virology, host genetics, and treatment over the past few decades.

Human genetics research involving the infectious disease HIV has progressed considerably after initiation of the human genome project (HGP), which is sequencing the entire human genome, both physically and functionally [1]. Many host genetic factors that influence AIDS epidemiological heterogeneity have been characterized [2–4]. From the HIV entry receptor on lymphoid cells to oncogenes in human glioblastomas, AIDS restriction genes (ARGs) are widely involved in biological pathways, and nearly 40 ARGs have been studied in depth through functional analyses [5–12]. Host genomic analysis is a key approach to studying AIDS epidemiology [13].

Further, genome, transcriptome, proteome, and metabolome biodatasets related to HIV have grown exponentially due to advanced sequencing technology. However, an integrative study on these datasets is limited in terms of understanding the complicated biological network.

Recent studies have revealed that metabolic pathways exert certain effects on the control of AIDS disease progression [14]. For example, the oxygen concentration can modulate T-cell differentiation through controlling metabolic status [15]. Metabolizing ATP to adenosine inhibits HIV-specific effector cells. Further, HIV infection is affected by dNTP hydrolysis. Efficient HIV-1 infection of CD4(+) lymphocytes requires sufficient glucose uptake via the Glut1 glucose transporter [16]. Tryptophan and phenylalanine metabolism also play an important role in HIV because HIV pathophysiology is associated with inflammatory stress due to dysregulated amino acid metabolism [17]. The HIV protein NEF impacts lipid-related metabolism through impairing cholesterol metabolism in both infected and bystander cells [18, 19]. This evidence suggests that cross talk between AIDS and the host metabolism is an important research topic that is necessary to resolve the disease mechanism and aid in therapy. Integrating biodatasets with an in-depth analysis of host AIDS restriction genes and metabolic pathways is imperative.

In the transcriptome, gene coexpression is a model for understanding how individual genes are correlated in certain conditions [20, 21]. Based on advances in this field, researchers hypothesize that the coexpression of genes in certain pathways indicates an integrative correlation between the two molecular pathways. Full genes in metabolic pathway are available for the human genome. Identifying correlations between a group of metabolic pathway genes and ARGs is a more comprehensive means for understanding integrative biodatasets. However, traditional methods using a Pearson or partial correlation are only suitable for a single gene. A canonical correlation analysis (CCA) is an efficient and powerful approach for measuring coexpression between two sets of genes. A Childhood Asthma Management Program (CAMP) study using a CCA successfully detected genetic regulatory variants [22]. Using the CCA, the glioblastoma transcriptomes of 45 patients were thoroughly analyzed to identify the glioma pathway genes [23].

In this paper, we used a CCA to analyze coexpression between ARGs and metabolic pathways from KEGG. We discuss the most important metabolic pathways coexpressed with the ARGs, which may imply strategies for AIDS diagnosis and therapy.

## 2. Methods

### 2.1. Datasets

Human genome expression datasets were downloaded from the website COPRESDB (http://coxpresdb.jp/), which contains approximately 4000 experiments and expression data on 20,000 human genes. Metabolic pathway genes were downloaded from KEGG (http://www.kegg.jp/); this dataset includes 129 typical metabolic pathways with predicted genes. The ARGs were collected from published literature. Two expression datasets were generated to include metabolic pathway gene and ARG expression data, respectively (Tables 1 and 2).

### 2.2. Canonical Correlation Analysis

To analyze the correlations between ARG and metabolic pathway gene expression, we used a CCA, which integrates multiple correlations into a few significant correlations. This statistical method calculates the correlation between two sets of variables and generates statistically independent pairs of new variables, which are referred to as canonical variables. The linear combination of the variables creates a component of the canonical variable pair in each group of the original variables.

In this study, these variables were defined at each flag as follows: ARG expression described by *M* genes in the vector *c* = (*c*_1_, *c*_2_,…, *c*_*M*_) and metabolic pathway gene expression described by *N* genes in the vector *k* = (*k*_1_, *k*_2_,…, *k*_*M*_). The respective sets of canonical variables *s* = (*s*_1_, *s*_2_,…, *s*_*M*_) and *p* = (*p*_1_, *p*_2_,…, *p*_*M*_) are results from the linear combination of ARG and metabolic pathway gene expression. The ARG expression canonical variables are included in the vector *s*, which is the result of the linear combination comprising the *c* vector (original ARGs expression) and the canonical coefficients vector as *s* = *A*′*c*. The vector contains the canonical variables for metabolic pathway gene expression, which result from the linear combination of the vector (original metabolic pathway genes expression) and canonical coefficient vector. The ARG and metabolic pathway gene variance-covariance matrices can be used to estimate the canonical correlation coefficients.

The magnitude of the correlation between each pair of canonical variables is described by the vector *k*_*i*_ eigenvalues. The canonical coefficients exist in the eigenvectors and can be used to estimate the canonical variables. The variance-covariance matrices contain the variances and covariances within the groups for the ARGs and metabolic pathway genes, respectively. The covariances between variables were calculated from the variance-covariance matrices.

### 2.3. The Study Design and Software Tools

The canonical correlation analysis was performed using the R platform (http://www.r-project.org/). After the canonical variables were generated from the expression datasets composed of ARGs and metabolic pathway genes, we set the absolute value 0.15 as the threshold for selecting ARGs correlated with canonical variables. To select metabolic pathway genes correlated with canonical variables, we sorted the genes using the absolute value, and the top 50 were selected for further enrichment analyses. Functional annotations were generated and enrichment analyses were performed for the metabolic pathway genes using the web-based DAVID tool (http://david.abcc.ncifcrf.gov/). For the pathway enrichment analyses, the “KEGG_PATHWAY” was selected. The pathways with a *P* value < 0.01 were considered significant.

## 3. Results

### 3.1. The ARGs and Metabolic Pathway Genes

#### 3.1.1. The General CCA Results

Eight significant (*P* < 0.01, Wilk's Lambda, *r* > 0.95) canonical correlations were discerned between the ARG and metabolic pathway gene transcriptomes using the CCA. 60% of the total ARG expression variance was explained by the ARGs canonical variables. Significant metabolic pathway canonical variables explained 38% of the metabolic gene transcriptome variation. Thus, ARG-metabolic pathway associations were involved in a substantial proportion of the total variance. The first pair of canonical variables had a correlation of 0.99, while the second pair of canonical variables had a correlation of 0.98.

### 3.2. Relationships between the Canonical Variables and Original Genes

#### 3.2.1. Pair 1 (*C*1,* P*1)

As shown in Table 3, the canonical variable* C*1 explains 2.4% of the variability in the original ARGs expression variables. We observed positive correlations (absolute value > 0.15) with all ARGs, including PPIA (0.42), ZNRD1 (0.37), MYH9 (0.36), TSG101 (0.31), IDH1 (0.28), TRIM5a (0.17), and CUL5 (0.15), but not GML (−0.17) and NCOR2 (−0.31). The greatest positive correlation was observed between *C*1 and PPIA. In contrast, the greatest negative correlation was observed between *C*1 and NCOR2. Among seven ARGs with positive correlations, the four ARGs, PPIA, TSG101, TRIM5a, and CUL5, are postentry cellular viral cofactors.

As shown in Table 4, the canonical variable *P*1 accounts for the variability in the original metabolic pathway gene expression data. The metabolic pathway genes that correlated with variable *P*1 were enriched for purine metabolism; these genes include phosphodiesterase 4C (5143), polymerase (RNA) III (DNA directed) polypeptide K (51728), and primase (5558).

#### 3.2.2. Pair 2 (*C*2,* P*2)

As shown in Table 3, the canonical variable *C*2 explains 5.3% of the variability in the original ARG expression variables. This variable highly correlated with the ARGs PPIA (0.92), CUL5 (0.51), TSG101 (0.48), IDH1 (0.17), and PECI (0.15), but not GML (−0.16), APOBEC3G (−0.17), MYH9 (−0.17), IL4 (−0.18), TLR9 (−0.18), CXCR1 (−0.25), HLA-C (−0.26), NCOR2 (−0.28), DC-SIGN (−0.29), and TLR8 (−0.36). The greatest positive correlation was observed between *C*2 and PPIA. However, the greatest negative correlation was observed between *C*2 and DC-SIGN. Among the ARGs with large correlations, PPIA, TSG101, CUL5, and APOBEC3G are postentry cellular viral cofactors. Among the ARGs with negative correlations, CXCR1 and IL4 are related to cytokines. DC-SIGN is involved in chemokines, which play important role in HIV entry through chemokine receptors.

As shown in Table 4, the canonical variable *P*2 accounts for the variability in the original metabolic pathway gene expression data. The metabolic pathway genes that highly correlate with the variable *P*2 are not enriched in a certain pathway.

#### 3.2.3. Pair 3 (*C*3,* P*3)

As shown in Table 3, the canonical variable *C*3 explains 12.7% of the variability on the original ARG expression variables. This variable positively correlated (absolute value > 0.15) with PPIA (1.88), NCOR2 (0.37), ZNRD1 (0.28), MYH9 (0.21), CXCR1 (0.20), and Slurp1 (0.19); in contrast, it negatively correlated with TRIM5a (−0.15), SDF1 (−0.17), IDH1 (−0.22), PECI (−0.24), TSG101 (−0.25), and CUL5 (−0.87). The greatest positive correlation was observed between *C*1 and PPIA. However, the greatest negative correlation was observed between *C*3 and CUL5. Among the ARGs that highly correlated with *C*3, PPIA, TSG101, TRIM5a, and CUL5 are postentry cellular viral cofactors. However, only PPIA positively correlated with *C*3.

As shown in Table 4, the canonical variable *P*3 accounts for the variability in the original metabolic pathway gene expression data. The metabolic pathway genes that highly correlated with the variable *P*3 are enriched in glycolysis and pyrimidine metabolism. The glycolysis genes include phosphoglycerate mutase 1 (5223), glyceraldehyde-3-phosphate dehydrogenase (2597), and glucose-6-phosphatase (57818). The pyrimidine metabolism genes include polymerase (DNA directed), delta 2 (5425), cytidine monophosphate (UMP-CMP) kinase 1 (51727), and uridine monophosphate synthetase (7372).

#### 3.2.4. Pair 4 (*C*4,* P*4)

As shown in Table 3, the canonical variable *C*4 explains 3.3% of the variability in the original ARG expression variables. This variable highly correlated (absolute value > 0.15) with PPIA (0.58), TLR8 (0.30), TLR4 (0.24), and PROX1 (0.16), but not DEFB1 (−0.15), IDH1 (−0.17), SDF1 (−0.18), CUL5 (−0.23), LY6D (−0.24), NCOR2 (−0.27), and Slurp1 (−0.61). The greatest positive correlation was observed between *C*4 and PPIA. However, the greatest negative correlation was observed between *C*4 and Slurp1. Among the ARGs that highly correlated with *C*3, only PPIA and CUL5 are postentry cellular viral cofactors.

As shown in Table 4, the canonical variable *P*4 accounts for the variability in the original metabolic pathway gene expression data. The metabolic pathway genes that highly correlated with the variable *P*4 are enriched in purine metabolism. These genes include deoxyguanosine kinase (1716), polymerase (RNA) III (DNA directed) polypeptide K (51728), polymerase (RNA) III (DNA directed) polypeptide B (55703), pyruvate kinase (5313), adenylate cyclase 10 (55811), phosphodiesterase 6D (5147), polymerase (DNA directed), delta 2 (5425), polymerase (RNA) II (DNA directed) polypeptide C (5432), and phosphodiesterase 5A (8654).

#### 3.2.5. Pair 5 (*C*5,* P*5)

As shown in Table 3, the canonical variable *C*5 explains 8.3% of the variability in the original ARG expression variables. This variable highly correlated (absolute value > 0.15) with IDH1 (0.60), TLR8 (0.33), ZNRD1 (0.32), TRIM5a (0.26), IRF1 (0.23), PROX1 (0.23), Slurp1 (0.21), HLA-C (0.21), GML (0.21), CUL5 (0.19), CXCR1 (0.17), TSG101 (0.17), APOBEC3B (0.15), TLR4 (−0.15), DC-SIGN (−0.17), SDF1 (−0.26), HLA-B (−0.41), MYH9 (−0.49), PPIA (−0.54), and NCOR2 (−1.10). The greatest positive correlations were observed between *C*5 and IDH1. However, the greatest negative correlations were observed between *C*5 and NCOR2. Among the ARGs that highly correlated with *C*5, PPIA, TSG101, APOBEC3B, TRIM5a, and CUL5 are postentry cellular viral cofactors. HLA-C and HLA-B are members of the HLA system. DC-SIGN and SDF1 are related to chemokines. CXCR1 is related to the cytokines pathway.

As shown in Table 4, the canonical variable *P*5 accounts for the variability in the original metabolic pathway gene expression data. The metabolic pathway genes that highly correlated with the variable *P*5 are enriched in inositol phosphate metabolism; these genes include synaptojanin 2 (8871), phospholipase C beta 2 (5330), and inositol-trisphosphate 3-kinase B (3707).

#### 3.2.6. Pair 6 (*C*6,* P*6)

As shown in Table 3, the canonical variable *C*6 explains 10.8% of the variability in the original ARG expression variables. This variable highly correlated (absolute value > 0.15) with PPIA (1.11), TLR8 (0.70), TSG101 (0.49), CUL5 (0.40), Slurp1 (0.32), TLR4 (0.26), HLA-B (0.25), CXCR6 (0.18), TLR9 (−0.17), IDH1 (−0.20), HLA-A (−0.22), IRF1 (−0.24), NCOR2 (−0.24), TRIM5a (−0.30), HLA-C (−0.33), CXCR1 (−0.40), MYH9 (−0.50), and ZNRD1 (−0.91). The greatest positive correlation was observed between *C*6 and PPIA. However, the greatest negative correlation was observed between *C*6 and ZNRD1. Among the ARGs that highly correlated with *C*6, PPIA, TSG101, TRIM5a, and CUL5 are postentry cellular viral cofactors. HLA-A, HLA-C, and HLA-B are members of the HLA system. CXCR6 is related to chemokine receptors. IRF1 and CXCR1 are related to cytokines.

As shown in Table 4, the canonical variable *P*6 accounts for the variability in the original metabolic pathway gene expression data. The metabolic pathway genes that highly correlated with variable *P*6 are enriched in pyrimidine metabolism and terpenoid backbone biosynthesis. These genes include polymerase (RNA) II (DNA directed) polypeptide F (5435), cytidine monophosphate (UMP-CMP) kinase 1 (51727), polymerase (RNA) I polypeptide B (84172), farnesyl diphosphate synthase (2224), and acetyl-CoA acetyltransferase 1 (38).

#### 3.2.7. Pair 7 (*C*7,* P*7)

As shown in Table 3, the canonical variable *C*7 explains 9% of the variability in the original ARG expression variables. This variable highly correlated (absolute value > 0.15) with IDH1 (1.12), PROX1 (0.55), CCL11 (0.25), DC-SIGN (0.22), TRIM5a (0.22), KIR (0.17), IL4 (−0.15), TLR8 (−0.15), HLA-C (−0.22), TLR9 (−0.24), PECI (−0.35), NCOR2 (−0.38), APOBEC3G (−0.44), TSG101 (−0.54), and CUL5 (−0.80). The greatest positive correlation was observed between *C*7 and IDH1. However, the greatest negative correlation was observed between *C*7 and CUL5. Among the ARGs that highly correlated with *C*7, TSG101, APOBEC3G, TRIM5a, and CUL5 are postentry cellular viral cofactors. KIR and HLA-C are in the HLA system. DC-SIGN and CCL11 are related to chemokine receptors. IL4 is related to cytokines.

As shown in Table 4, the canonical variable *P*7 accounts for the variability in the original metabolic pathway gene expression data. The metabolic pathway genes that highly correlated with variable *P*7 are enriched in pyrimidine metabolism and methane metabolism. These genes include uridine-cytidine kinase 1-like 1 (54963), polymerase (RNA) II (DNA directed) polypeptide F (5435), polymerase (RNA) II (DNA directed) polypeptide A (5430), alcohol dehydrogenase 5 (class III) (128), and methylenetetrahydrofolate reductase (4524).

#### 3.2.8. Pair 8 (*C*8,* P*8)

As shown in Table 3, the canonical variable *C*8 explains 12% of the variability in the original ARG expression variables. This variable highly correlated (absolute value > 0.15) with PPIA (1.12), IDH1 (0.63), TRIM5a (0.53), NCOR2 (0.52), APOBEC3G (0.39), TLR9 (0.36), DC-SIGN (0.33), IL4 (0.30), PECI (0.25), SDF1 (0.22), TLR8 (0.17), MYH9 (−0.19), HLA-C (−0.21), IRF1 (−0.24), HLA-B (−0.31), PROX1 (−0.66), and TSG101 (−1.03). The greatest positive correlation was observed between *C*8 and PPIA. However, the greatest negative correlation was observed between *C*8 and TSG101. Among the ARGs that highly correlated with *C*8, TSG101, APOBEC3G, TRIM5a, and PPIA are postentry cellular viral cofactors. KIR and HLA-C are in the HLA system. DC-SIGN and SDF1 are related to chemokine receptors. IL4 and IRF1 are related to cytokines. HLA-C and HLA-B are in the HLA system.

As shown in Table 4, the canonical variable *P*8 accounts for the variability in the original metabolic pathway gene expression data. The metabolic pathway genes that highly correlated with variable *P*8 are not enriched in a metabolic pathway.

## 4. Discussion

Researchers have used numerous approaches to identify host genes related to AIDS [5–13]. Most studies use genomic information but not integration of the genome and transcriptome. However, most SNPs at ARGs impact AIDS through changing host gene transcription [7–10]. This study features novel experiments that focus on ARG cooperation at the transcription level and extends the correlation between ARGs and metabolic pathway genes to discover novel host genes related to AIDS.

For each variable in the canonical correlation analysis, HIV-1 postentry cellular viral cofactors highly cooperated at the transcription level. PPIA, TSG101, TRIM5a, APOBEC3G, and CUL5 frequently appeared together to correlate with the canonical variables. PPIA functions in cyclosporin A-mediated immunosuppression by encoding a member of the peptidyl-prolyl cis-trans isomerase (PPIase) family [24]. Formation of HIV virions requires an interaction between PPIA and HIV viral proteins. TSG101 negatively regulates cell growth and differentiation by producing a protein that interacts with stathmin [25]. TRIM5a is an E3 ubiquitin-ligase, and its ubiquitination function is involved in retroviral restriction [26]. These genes encode HIV-1 postentry cellular viral cofactors involved in different biological processes. Thus, the high correlation between these genes and canonical variables demonstrates that these genes are coordinated at the transcriptional level. These data suggest that a potential transcriptional regulator for these genes may be a key host factor related to AIDS.

The high-frequency ARGs that correlated with canonical variables include PPIA, TSG101, CUL5, NCOR2, IDH1, and MYH9. PPIA, TSG101, and CUL5 are discussed above. NCOR2 with histone deacetylases is a nuclear receptor corepressor [27]. IDH1 encodes isocitrate dehydrogenases involved in cytoplasmic NADPH production and pyruvate metabolism [28]. MYH9 aids in maintaining cell shape, cell motility, and cytokinesis as a conventional nonmuscle myosin [29]. These ARGs are not enriched in a certain biological process. However, many host genetic factors have not been studied.

The low-frequency ARGs that correlated with canonical variables include DEFB1 with *C*4, KIR with *C*7, HLA-A with *C*5, CCL11 with *C*7, LY6D with *C*4, APOBEC3B with *C*5, and CXCR6 with *C*6. DEFB1 is a defensin and is implicated in cystic fibrosis pathogenesis [30]. HLA-A is a major histocompatibility complex class I heavy chain paralogue; these paralogues are expressed in nearly all cells [31]. CCL11 is chemokine (C-C motif) ligand 11 and is implicated in immunoregulatory and inflammatory processes [32]. CXCR6 is chemokine (C-X-C motif) receptor [33]. LY6D is a member of the lymphocyte antigen 6 complex [34]. APOBEC3B is a member of the cytidine deaminase gene family. Recent studies have revealed that these ARGs may be RNA-editing enzymes that control the cell cycle [35]. Further, these genes only correlated with one canonical variable, which suggests that the specificity of the correlation may determine the canonical variable correlated with a certain metabolic pathway.

The most significant metabolic pathway in our analysis is purine metabolism, which featured correlations with two canonical variables and the lowest *P* values. Recent studies analyzed purine codon patterns in variable and constant regions of HIV-1 and showed that HIV-1 RNA exhibits extreme enrichment in the purine A compared with most organisms [36]. These data suggest that a potential therapeutic agent against HIV-1 may involve novel purine derivatives [37]. Studies have elucidated twenty-four purine derivatives that act as HIV-1 Tat TAR interaction inhibitors [38]. More recently, research revealed that host cells with a modified purine biosynthesis pathway exhibit increased activity by tenofovir against sensitive and drug resistant HIV-1 [39]. In this study, we show a high correlation between ARG and purine metabolism gene expression. These data imply that purine metabolism genes are significant candidates for studying the host genomic or transcriptome influence on AIDS.

## 5. Conclusions

In this study, we used a CCA to analyze the correlations between ARG and metabolic pathway gene expression. The results show that HIV-1 postentry cellular viral cofactors are highly coexpressed, which suggests that regulating this group of host genes may be a key factor in studies to understand the AIDS-host interaction mechanism. Furthermore, we show that purine metabolism pathway genes coordinate with ARGs; this novel discovery supports future studies on AIDS therapy using purine derivatives. Both coexpressed ARGs and metabolic pathway genes also provide a new marker for AIDS diagnosis.

## Figures and Tables

**Table 1:** HIV host genetic factor genes.

Gene symbol	Gene ID	Effect
APOBEC3B	9582	Increase infection
APOBEC3G	60489	Accelerates AIDS
CCL11	6356	
CCL17	6361	
CCL18	6362	
CCL2	6347	
CCL4	6351	
CCL5	6352	
CUL5	8065	Accelerates CD4 loss
CXCR1	3577	
CXCR6	10663	Accelerates AIDS
DC-SIGN	30835	Decreases infection
DEFB1	1672	
GML	2765	
HCP5	10866	HIV set point
HLA-A	3105	Delays AIDS
HLA-B	3106	Delays AIDS
HLA-C	3107	Delays AIDS
IDH1	3417	Prevents infection
IFENG	3458	Accelerates AIDS
IL10	3586	Accelerates AIDS
IL4	3565	
IRF1	3659	
KIR	2669	Delays AIDS
LY6D	8581	
MYH9	4627	End stage renal disease
NCOR2	9612	Increase infection
PECI/ECI2	10455	Accelerates AIDS
PPIA/CypA	5478	Accelerates AIDS
PROX1	5629	Delays AIDS progression
SDF1/CXCL12	6387	Delays AIDS
Slurp1	57152	
Slurp2/Ly6	6004	
TLR4	7099	
TLR8	51311	
TLR9	54106	
TRIM5a	85363	Increase infection
TSG101	7251	Accelerates AIDS
ZNRD1	30834	

**Table 2:** Human metabolism pathway for KEGG.

Pathway name	KEGG ID	Class of metabolism pathway	Gene number
Glycolysis/gluconeogenesis	10	Carbohydrate metabolism	67
Citrate cycle (TCA cycle)	20	Carbohydrate metabolism	31
Pentose phosphate pathway	30	Carbohydrate metabolism	29
Pentose and glucuronate interconversions	40	Carbohydrate metabolism	34
Fructose and mannose metabolism	51	Carbohydrate metabolism	36
Galactose metabolism	52	Carbohydrate metabolism	30
Ascorbate and aldarate metabolism	53	Carbohydrate metabolism	27
Starch and sucrose metabolism	500	Carbohydrate metabolism	56
Amino sugar and nucleotide sugar	520	Carbohydrate metabolism	49
Pyruvate metabolism	620	Carbohydrate metabolism	42
Glyoxylate and dicarboxylate metabolism	630	Carbohydrate metabolism	24
Propanoate metabolism	640	Carbohydrate metabolism	32
Butanoate metabolism	650	Carbohydrate metabolism	29
Inositol phosphate metabolism	562	Carbohydrate metabolism	61
Oxidative phosphorylation	190	Energy metabolism	133
Nitrogen metabolism	910	Energy metabolism	27
Sulfur metabolism	920	Energy metabolism	18
Fatty acid biosynthesis	61	Lipid metabolism	6
Fatty acid elongation	62	Lipid metabolism	23
Fatty acid metabolism	71	Lipid metabolism	44
Ketone bodies	72	Lipid metabolism	9
Steroid biosynthesis	100	Lipid metabolism	18
Primary bile acid biosynthesis	120	Lipid metabolism	17
Steroid hormone biosynthesis	140	Lipid metabolism	56
Glycerolipid metabolism	561	Lipid metabolism	55
Glycerophospholipid metabolism	564	Lipid metabolism	91
Ether lipid metabolism	565	Lipid metabolism	42
Sphingolipid metabolism	600	Lipid metabolism	47
Arachidonic acid metabolism	590	Lipid metabolism	68
Linoleic acid metabolism	591	Lipid metabolism	33
Alpha-linolenic acid metabolism	592	Lipid metabolism	25
Biosynthesis of unsaturated fatty acids	1040	Lipid metabolism	21
Purine metabolism	230	Nucleotide metabolism	173
Pyrimidine metabolism	240	Nucleotide metabolism	107
Alanine, aspartate, and glutamate metabolism	250	Amino acid metabolism	32
Glycine, serine, and threonine metabolism	260	Amino acid metabolism	37
Cysteine and methionine metabolism	270	Amino acid metabolism	34
Valine, leucine, and isoleucine degradation	280	Amino acid metabolism	44
Valine, leucine, and isoleucine biosynthesis	290	Amino acid metabolism	2
Lysine biosynthesis	300	Amino acid metabolism	2
Lysine degradation	310	Amino acid metabolism	49
Arginine and proline metabolism	330	Amino acid metabolism	57
Histidine metabolism	340	Amino acid metabolism	28
Tyrosine metabolism	350	Amino acid metabolism	39
Phenylalanine metabolism	360	Amino acid metabolism	18
Tryptophan metabolism	380	Amino acid metabolism	40
Phenylalanine, tyrosine, and tryptophan biosynthesis	400	Amino acid metabolism	5
Beta-alanine metabolism	410	Metabolism of other amino acids	29
Taurine and hypotaurine metabolism	430	Metabolism of other amino acids	10
Selenocompound metabolism	450	Metabolism of other amino acids	17
Cyanoamino acid metabolism	460	Metabolism of other amino acids	7
D-Glutamine and D-glutamate metabolism	471	Metabolism of other amino acids	4
D-Arginine and D-ornithine metabolism	472	Metabolism of other amino acids	1
Glutathione metabolism	480	Metabolism of other amino acids	51
N-Glycan biosynthesis	510	Glycan biosynthesis and metabolism	49
Mucin type O-glycan biosynthesis	512	Glycan biosynthesis and metabolism	31
Other types of O-glycan biosynthesis	514	Glycan biosynthesis and metabolism	30
Glycosaminoglycan biosynthesis, chondroitin sulfate/dermatan sulfate	532	Glycan biosynthesis and metabolism	20
Glycosaminoglycan biosynthesis, heparan sulfate/heparin	534	Glycan biosynthesis and metabolism	24
Glycosaminoglycan biosynthesis, keratan sulfate	533	Glycan biosynthesis and metabolism	15
Glycosaminoglycan degradation	531	Glycan biosynthesis and metabolism	19
Glycosylphosphatidylinositol- (GPI-) anchor biosynthesis	563	Glycan biosynthesis and metabolism	25
Glycosphingolipid biosynthesis, lacto- and neolactoseries	601	Glycan biosynthesis and metabolism	26
Glycosphingolipid biosynthesis, globoseries	603	Glycan biosynthesis and metabolism	14
Glycosphingolipid biosynthesis, ganglioseries	604	Glycan biosynthesis and metabolism	15
Other glycan degradation	511	Glycan biosynthesis and metabolism	18
Thiamine metabolism	730	Metabolism of cofactors and vitamins	4
Riboflavin metabolism	740	Metabolism of cofactors and vitamins	13
Vitamin B6 metabolism	750	Metabolism of cofactors and vitamins	6
Nicotinate and nicotinamide metabolism	760	Metabolism of cofactors and vitamins	28
Pantothenate and CoA biosynthesis	770	Metabolism of cofactors and vitamins	17
Biotin metabolism	780	Metabolism of cofactors and vitamins	3
Lipoic acid metabolism	785	Metabolism of cofactors and vitamins	3
Folate biosynthesis	790	Metabolism of cofactors and vitamins	14
One carbon pool by folate	670	Metabolism of cofactors and vitamins	20
Retinol metabolism	830	Metabolism of cofactors and vitamins	68
Porphyrin and chlorophyll metabolism	860	Metabolism of cofactors and vitamins	43
Ubiquinone and other terpenoid-quinone biosynthesis	130	Metabolism of cofactors and vitamins	10
Terpenoid backbone biosynthesis	900	Metabolism of terpenoids and polyketides	21
Caffeine metabolism	232	Biosynthesis of other secondary metabolites	7
Butirosin and neomycin biosynthesis	524	Biosynthesis of other secondary metabolites	5
Metabolism of xenobiotics by cytochrome P450	980	Xenobiotics biodegradation and metabolism	80
Drug metabolism, cytochrome P450	982	Xenobiotics biodegradation and metabolism	74
Drug metabolism, other enzymes	983	Xenobiotics biodegradation and metabolism	51

**Table 3:** Cross-correlation of Hf genes with canonical variate.

Gene symbol	*C*1	*C*2	*C*3	*C*4	*C*5	*C*6	*C*7	*C*8
DEFB1	0.01	0.01	0.02	−**0.15**	0.06	−0.02	−0.14	−0.04
KIR	0.08	−0.02	−0.05	−0.01	−0.14	0.08	**0.17**	−0.12
GML	−**0.17**	−**0.16**	0.12	−0.06	**0.21**	−0.06	0.03	0.07
HLA-A	0.10	−0.14	−0.01	0.00	−0.03	−**0.22**	−0.13	−0.09
HLA-B	0.10	0.09	0.12	0.12	−**0.41**	**0.25**	0.07	−**0.31**
HLA-C	0.07	−**0.26**	−0.04	−0.08	**0.21**	−**0.33**	−**0.22**	−**0.21**
IDH1	**0.28**	**0.17**	−**0.22**	−**0.17**	**0.60**	−**0.20**	**1.12**	**0.63**
IFENG	0.00	0.03	0.08	0.07	0.05	−0.09	−0.12	−0.07
IL4	−0.12	−**0.18**	0.08	0.05	0.01	−0.10	−**0.15**	**0.30**
CXCR1	−0.07	−**0.25**	**0.20**	0.00	**0.17**	−**0.40**	−0.14	0.08
IL10	−0.02	−0.05	0.02	0.13	0.05	−0.04	−0.05	−0.01
IRF1	0.07	−0.09	0.08	0.10	**0.23**	−**0.24**	−0.14	−**0.24**
MYH9	**0.36**	−**0.17**	**0.21**	−0.14	−**0.49**	−**0.50**	0.14	−**0.19**
PPIA/CypA	**0.42**	**0.92**	**1.88**	**0.58**	−**0.54**	**1.11**	0.00	**1.12**
PROX1	−0.14	0.07	0.03	**0.16**	**0.23**	0.02	**0.55**	−**0.66**
Slurp2/Ly6	−0.04	−0.03	0.00	0.10	−0.13	0.12	0.09	0.02
CCL2	0.02	−0.03	−0.04	0.00	−0.05	0.12	0.08	0.00
CCL4	0.02	−0.06	0.02	0.09	0.02	0.05	0.04	−0.06
CCL5	0.03	−0.06	0.01	0.03	0.00	0.02	−0.14	0.05
CCL11	0.02	−0.05	0.01	0.02	0.09	−0.01	**0.25**	−0.05
CCL17	0.00	−0.06	0.05	0.07	0.06	−0.02	−0.19	−0.06
CCL18	0.03	−0.04	0.00	0.06	0.05	0.14	0.09	0.05
SDF1/CXCL12	0.09	−0.10	−**0.17**	−**0.18**	−**0.26**	0.14	0.09	**0.22**
TLR4	0.06	−0.05	−0.02	**0.24**	−**0.15**	**0.26**	0.00	0.02
TSG101	**0.31**	**0.48**	−**0.25**	−0.05	**0.17**	**0.49**	−**0.54**	−**1.03**
CUL5	**0.15**	**0.51**	−**0.87**	−**0.23**	**0.19**	**0.40**	−**0.80**	−0.04
LY6D	0.01	−0.05	0.10	−**0.24**	0.10	0.13	0.01	−0.08
APOBEC3B	0.04	0.03	0.04	0.03	**0.15**	−0.12	0.02	0.14
NCOR2	−**0.31**	−**0.28**	**0.37**	−**0.27**	−**1.10**	−**0.24**	−**0.38**	**0.52**
PECI/ECI2	0.09	**0.15**	−**0.24**	0.01	−0.10	0.06	−**0.35**	**0.25**
CXCR6	−0.06	−0.10	0.02	−0.06	0.03	**0.18**	−0.07	0.09
HCP5	−0.04	0.02	−0.01	−0.01	0.32	0.01	0.02	−0.01
ZNRD1	**0.37**	0.14	**0.28**	−0.03	**0.32**	−**0.91**	−0.04	0.10
DC-SIGN	−0.04	−**0.29**	0.13	0.00	−**0.17**	0.03	**0.22**	**0.33**
TLR8	0.13	−**0.36**	−0.03	**0.30**	**0.33**	**0.70**	**−0.15**	**0.17**
TLR9	−0.03	−**0.18**	0.11	−0.06	−0.02	−**0.17**	**−0.24**	**0.36**
Slurp1	−0.03	−0.10	**0.19**	−**0.61**	**0.21**	**0.32**	0.04	−0.06
APOBEC3G	0.06	−**0.17**	−0.14	0.11	0.19	0.07	−**0.44**	**0.39**
TRIM5a	**0.17**	−0.13	−**0.15**	0.00	**0.26**	−**0.30**	**0.22**	**0.53**

**Table 4:** Cross-correlation of genes enriched in metabolic pathways with canonical variate.

Component	Term	Count	Pop hits	*P* value	Genes
*P*1+	Purine metabolism	3	153	4.87*E* − 02	5143, 51728, 5558
*P*3+	Glycolysis/gluconeogenesis	3	60	1.14*E* − 02	5223, 2597, 57818
*P*3+	Pyrimidine metabolism	3	95	2.72*E* − 02	5425, 51727, 7372
*P*4−	Purine metabolism	4	153	7.62*E* − 03	1716, 51728, 55703, 5313
*P*4+	Purine metabolism	5	153	6.23*E* − 04	55811, 5147, 5425, 5432, 8654
*P*5−	Inositol phosphate metabolism	3	54	6.82*E* − 03	8871, 5330, 3707
*P*6−	Pyrimidine metabolism	3	95	2.72*E* − 02	5435, 51727, 84172
*P*6+	Pyruvate metabolism	3	40	3.17*E* − 03	5162, 4191, 38
*P*6+	Terpenoid backbone biosynthesis	2	15	3.20*E* − 02	2224, 38
*P*7−	Pyrimidine metabolism	3	95	2.72*E* − 02	54963, 5435, 5430
*P*7+	Methane metabolism	2	6	1.52*E* − 02	128, 4524
